# The downregulation of miR-509-3p expression by collagen type XI alpha 1-regulated hypermethylation facilitates cancer progression and chemoresistance via the DNA methyltransferase 1/Small ubiquitin-like modifier-3 axis in ovarian cancer cells

**DOI:** 10.1186/s13048-023-01191-5

**Published:** 2023-06-29

**Authors:** Yi-Hui Wu, Yu-Fang Huang, Pei-Ying Wu, Tzu-Hao Chang, Soon-Cen Huang, Cheng-Yang Chou

**Affiliations:** 1 Department of Medical Research, Chi Mei Medical Center, Liouying Tainan, 73657 Taiwan; 2 Department of Nursing, Min-Hwei Junior College of Health Care Management, Tainan, 73658 Taiwan; 3 Department of Obstetrics and Gynecology, College of Medicine, National Cheng Kung University Hospital, National Cheng Kung University, 70403 Tainan, Taiwan; 4 Graduate Institute of Biomedical Informatics, Taipei Medical University, Taipei, 110 Taiwan; 5 Department of Obstetrics and Gynecology, Chi Mei Medical Center, Liouying, Tainan, 73657 Taiwan

**Keywords:** Epithelial ovarian carcinoma, Collagen type XI alpha 1, miR-509-3p, DNMT1

## Abstract

**Background:**

MicroRNAs are a group of small non-coding RNAs that are involved in development and diseases such as cancer. Previously, we demonstrated that miR-335 is crucial for preventing collagen type XI alpha 1 (COL11A1)-mediated epithelial ovarian cancer (EOC) progression and chemoresistance. Here, we examined the role of miR-509-3p in EOC.

**Methods:**

The patients with EOC who underwent primary cytoreductive surgery and postoperative platinum-based chemotherapy were recruited. Their clinic-pathologic characteristics were collected, and disease-related survivals were determined. The COL11A1 and miR-509-3p mRNA expression levels of 161 ovarian tumors were determined by real-time reverse transcription-polymerase chain reaction. Additionally, miR-509-3p hypermethylation was evaluated by sequencing in these tumors. The A2780CP70 and OVCAR-8 cells transfected with miR-509-3p mimic, while the A2780 and OVCAR-3 cells transfected with miR-509-3p inhibitor. The A2780CP70 cells transfected with a small interference RNA of COL11A1, and the A2780 cells transfected with a COL11A1 expression plasmid. Site-directed mutagenesis, luciferase, and chromatin immunoprecipitation assays were performed in this study.

**Results:**

Low miR-509-3p levels were correlated with disease progression, a poor survival, and high COL11A1 expression levels. In vivo studies reinforced these findings and indicated that the occurrence of invasive EOC cell phenotypes and resistance to cisplatin are decreased by miR-509-3p. The miR-509-3p promoter region (p278) is important for miR-509-3p transcription regulation via methylation. The miR-509-3p hypermethylation frequency was significantly higher in EOC tumors with a low miR-509-3p expression than in those with a high miR-509-3p expression. The patients with miR-509-3p hypermethylation had a significantly shorter overall survival (OS) than those without miR-509-3p hypermethylation. Mechanistic studies further indicated that miR-509-3p transcription was downregulated by COL11A1 through a DNA methyltransferase 1 (DNMT1) stability increase. Moreover, miR-509-3p targets small ubiquitin-like modifier (SUMO)-3 to regulate EOC cell growth, invasiveness, and chemosensitivity.

**Conclusion:**

The miR-509-3p/DNMT1/SUMO-3 axis may be an ovarian cancer treatment target.

**Supplementary Information:**

The online version contains supplementary material available at 10.1186/s13048-023-01191-5.

## Background

Epithelial ovarian cancer (EOC) is a highly lethal and heterogeneous disease characterized by a distinctive propensity for peritoneal spread, whereas metastasis to distant organs only tends to occur in the late stages [1]. The standard EOC treatment includes cytoreductive surgery followed by combination chemotherapy with carboplatin and paclitaxel; however, some patients relapse due to chemoresistance, and can even die from the disease [2]. Therefore, it is important to identify markers that predict the patient outcome, which may enable the development of prognostic or therapeutic biomarkers.

MicroRNAs (miRNAs), a class of small noncoding RNAs (approximately 22 nt), have a post-transcriptional regulation function of binding to the 3′-untranslated region (3′-UTR) of target mRNAs, resulting in mRNA degradation or translation inhibition [3–6]. An altered miRNA expression has been reported in almost all human cancer types. miRNAs can function as oncogenes or tumor suppressor genes, being involved in multiple pathways and cell functions related to cancer development and progression, such as proliferation, apoptosis, invasion, and resistance to therapy [3–8]. Several studies have shown that some miRNAs are potential diagnostic and prognostic markers in ovarian cancer [3–6].

Previous reports have indicated that microRNA-509 (miR-509-3p) is a strong tumor suppressor that decreases the migration of multiple ovarian cancer cell lines and disrupts their multi-cellular spheroids [9]. miR-509-3p overexpression not only downregulates the expression of X-linked inhibitor of apoptosis protein (XIAP) in ovarian cancer cells, but also inhibits the proliferation of EOC cells and increases their sensitivity to cisplatin-induced apoptosis [10, 11]. However, the mechanisms underlying miR-509-3p expression regulation in ovarian cancer remain poorly understood.

Collagen type XI alpha 1 (COL11A1), belonging to the collagen family, is the most abundant component of the interstitial extracellular matrix. COL11A1 expression is upregulated in several cancer types, including ovarian, breast, pancreatic, non-small-cell lung, and colorectal cancer. High COL11A1 levels are associated with tumor aggressiveness, chemoresistance, and a poor survival [12–18]. Moreover, COL11A1 can serve as a specific biomarker for cancer-associated fibroblasts (CAFs) [19–21] and as a novel therapeutic target for cancer treatment [22, 23].

We have previously identified miR-509-3p and miR-335 as candidate miRNAs that regulate COL11A1 expression, through an online database search. We demonstrated the importance of miR-335 in decreasing the occurrence of COL11A1-mediated ovarian tumor progression, chemoresistance, and the likelihood of poor survival [24]. Interestingly, controlling the miR-509 level did not directly regulate the mRNA expression of COL11A1. However, miR-509-3p expression was positively correlated with the COL11A1 level in EOC tumor samples and cell lines. These results suggest a link between miR-509-3p and COL11A1. In this study, we showed that miR-509-3p acts as a novel methylation-responsive tumor suppressor in ovarian cancer. The tumor-suppressive function of miR-509-3p is mediated, at least in part, by the suppression of the small ubiquitin-like modifier (SUMO)-3.

## Results

### A low miR-509-3p level correlates with a poor clinical outcome

**Fig. 1:**
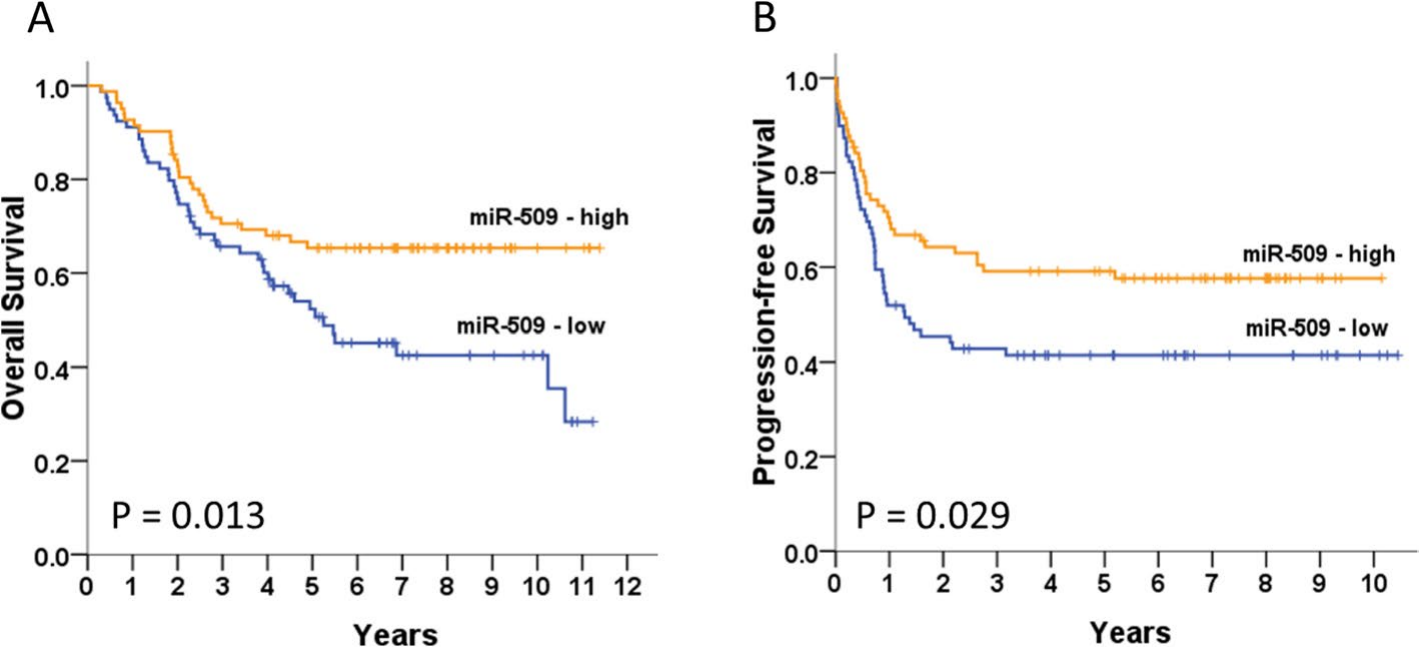
Ten-year overall survival (OS) (**A**) and progression-free survival (PFS) (**B**). Kaplan–Meier curves stratified by the miR-509-3p mRNA level and analyzed using a log-rank test

To investigate the expression level and significance of miR-509-3p in ovarian cancer, we first performed quantitative real-time PCR to evaluate the miR-509-3p expression level in 161 ovarian cancer and 23 non-cancerous tissues. Low miR-509-3p mRNA levels in patients with EOC were significantly associated with old age (*P* = 0.005), an advanced stage (*P* < 0.001), serous histology (*P* = 0.002), and cancer death (*P* = 0.010) (Table 1). However, no correlation was found between the miR-509-3p levels and the response to chemotherapy (*P* = 0.188) or the progression-free interval (*P* = 0.286). The miR-509-3p levels in cancerous tissues tended to be lower than those in non-cancerous tissues (Supplementary Table 1). Furthermore, low miR-509-3p levels were significantly related to high COL11A1-expressing tumors (*P* < 0.001). Patients with low miR-509-3p mRNA levels have significantly shorter OS (Fig. 1A, *P* = 0.013) and PFS (Fig. 1B, *P*  = 0.029) than those with high miR-509-3p levels.

**Table 1:** EOC patient characteristics and the studied biomarkers (*n* = 161)

	N	miR-509-3p		*P*
		Low	High	
Age				0.005
< 52	67	24 (35.8)	43 (64.2)	
≥ 52	94	55 (58.5)	39 (41.5)	
Stage				< 0.001
I-II	73	19 (26.0)	54 (74.0)	
III-IV	88	60 (68.2)	28 (31.8)	
Histology				0.002
Serous	94	56 (59.6)	38 (40.4)	
Non-serous	67	23 (34.3)	44 (65.7)	
Mucinous		0 (0)	11 (100)	
Endometrioid		7 (63.6)	4 (36.4)	
Clear cell		16 (35.6)	29 (64.4)	
Chemotherapy	136			0.188
CR/PR	99	49 (49.5)	50 (50.5)	
SD/PD	37	23 (62.2)	14 (37.8)	
PFI				0.286
≥ 6 m	124	58 (46.8)	66 (53.2)	
< 6 m	37	21 (56.8)	16 (43.2)	
Death				0.010
No	90	36 (40.0)	54 (60.0)	
Yes	71	43 (60.6)	28 (39.4)	

Data was presented as frequency (percentage)

Data was analyzed by Chi-square test or Fisher’s exact test

miR-509-3p level < 0.76 (low) and ≥ 0.76 (high); COL11A1 level < 1446.67 (low) and ≥ 1446.67 (high)

### The invasive phenotypes of EOC cells are regulated by miR-509-3p

**Fig. 2:**
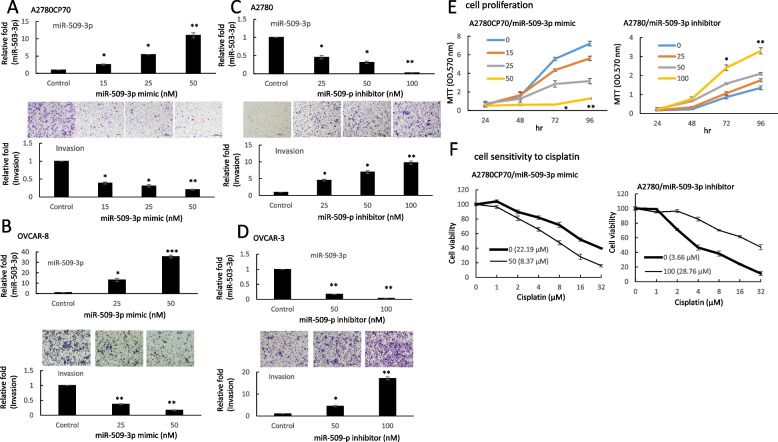
The invasive phenotypes of EOC cells are regulated by miR-509-3p. **A** and **B** Upper panel: A2780CP70 and OVCAR-8 cells were transfected with miR-509-3p mimics and miR-509-3p expression levels were measured using real-time reverse transcription-polymerase chain reaction (RT-PCR) in were evaluated. Lower panel: A2780CP70 and OVCAR-8 cells were transfected with miR-509-3p mimics for 48 h; representative images of cell invasion is shown. All data represent the mean ± SD of experimental triplicates; * *P* < 0.05, ** *P* < 0.01, *** *P* < 0.001, compared with the control. **C** and **D** Upper panel: A2780 and OVCAR-3 cells were transfected with a miR-509-3p inhibitor and miR-509-3p expression levels were measured using real-time RT-PCR. Lower panel: A2780 and OVCAR-3 cells were transfected with a miR-509-3p inhibitor for 48 h; representative images of cell invasion is shown. All data represent the mean ± SD of experimental triplicates; * *P* < 0.05, ** *P* < 0.01, compared with the control. **E** Proliferation of A2780CP70 and A2780 cells transfected with miR-509-3p mimics and the miR-509-3p inhibitor, respectively, for 48 h. All experiments were performed in triplicate; with the corresponding * *P* < 0.05, ** *P *< 0.01, compared with the control. **F** A2780CP70 and A2780 cells were transfected with miR-509-3p mimics and the miR-509-3p inhibitor, respectively, for 48 h. Following transfection, cells were treated with different concentrations of cisplatin for 48 h. 3-(4,5-Dimethylthiazol-2-yl)-2,5-diphenyltetrazolium bromide assays were performed to confirm cell viability in all assays E & F. All experiments were performed in triplicate

To determine the impact of miR-509-3p on the aggressive traits of EOC cells, A2780CP70 and OVCAR-8 cells were transfected with miR-509-3p mimics to increase the expression of miR-509-3p (Figs. 2A and B, lower panel), and A2780 and OVCAR-3 cells were transfected with the miR-509-3p inhibitor to decrease the expression of miR-509-3p (Figs. 2C and 2D, lower panel). Transwell invasion assays revealed a lower number of invading cells in the miR-509-3p mimic-transfected cells (Figs. 2A and B, upper panel), whereas the invading cell number increased in the miR-509-3p inhibitor-transfected cells (Figs. 2C and D, upper panel). The cell viabilities of miR-509-3p mimic-transfected cells were significantly decreased (Fig. 2E, left panel) and those of miR-509-3p inhibitor-transfected cells were increased (Fig. 2E, right panel). We then examined the chemoresistance regulation by miR-509-3p in EOC cells. The cell sensitivity to cisplatin increased in the miR-509-3p mimic-transfected A2780CP70 cells (IC_50_ value: from 22.19 to 8.37 μM, *P* < 0.01, Fig. 2F, upper panel), whereas it decreased in the miR-509-3p inhibitor-transfected A2780 cells (IC_50_ value: from 3.66 to 28.76 μM, *P* < 0.01, Fig. 2F, lower panel) in a dose-dependent manner. These results indicate that the aggressive phenotypes and cisplatin resistance are reversed by miR-509-3p in ovarian cancer cells.

### Hypermethylation of the p278 promoter region regulates miR-509-3p transcription

**Fig. 3:**
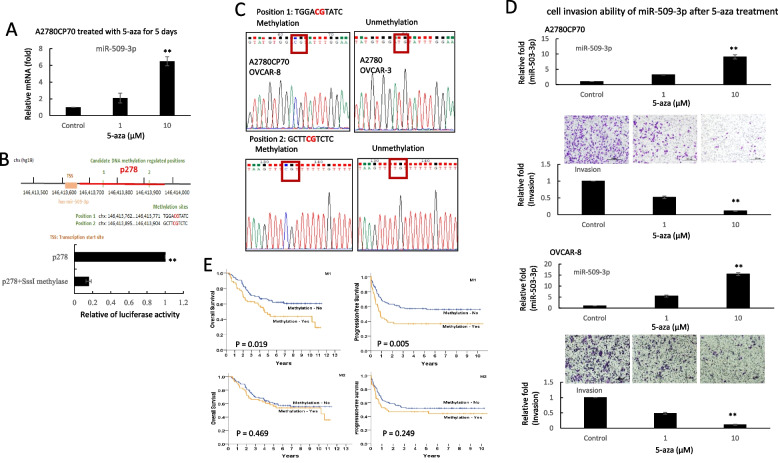
Hypermethylation of the p278 promoter region regulates miR-509-3p transcription. **A** mRNA expression levels of miR-509-3p in A2780CP70 cells treated with 1 or 10 µM 5-aza-2′-deoxycytidine (5-aza) for 5 days were evaluated using real-time RT-PCR. All experiments were performed in triplicate. ** *P* < 0.01, compared with the control. **B** Upper panel: The genomic position of miR-509-3p and two candidate methylation-regulated positions (p278: chr146,413,629 to 146,413,907) are shown. Lower panel: A2780CP70 cells were transfected with SssI methylated or unmethylated p278. Luciferase activity was almost null in the cells transfected with SssI methylated p278. All experiments were performed in triplicate; ** *P* < 0.01. **C** Representative bisulfite sequencing. **D** The invasive ability of A2780CP70 and OVCAR-8 cells treated with 5-aza (1 or 10 μM) for 5 days. All data represent the mean ± SD of three separate experiments; ** *P* < 0.01, compared with the control. **E** Ten-year overall survival (OS) and progression-free survival (PFS). Kaplan–Meier curves stratified by the miR-509-3p promoter methylation at position 1 (upper panel) and at position 2 (lower panel), analyzed using a log-rank test (*n* = 161)

Several studies have shown that certain miRNA genes are silenced in human tumors via aberrant CpG island hypermethylation [25]. To understand whether miR-509-3p is downregulated by hypermethylation in ovarian cancer tumorigenesis, A2780CP70 cells were treated with the demethylation reagent 5-aza. The expression of miR-509-3p was restored after 5-aza treatment (Fig. 3A). The promoter region information of the miR-509-3p gene was obtained from FANTOM database [26], and we identified two CpG sites in this region (Fig. 3B, upper panel). To examine whether this region could attenuate transcription upon hypermethylation, p278 (chr 146,413,629 to 146,413,907) was methylated by SssI methylase and transfected into A2780CP70 cells. Luciferase activity was almost null in these cells (Fig. 3B, lower panel). These results suggest that the methylation of the two CpG sites in miR-509-3p is responsible for promoter downregulation.

To further confirm that the promoter hypermethylation of miR-509-3p was the main mechanism responsible for the decrease in the expression of miR-509-3p, four ovarian cell lines were sequenced. Sequencing analysis revealed that miR-509-3p methylation was observed in A2780CP70 and OVCAR-8 cells, which have a low miR-509-3p expression. Conversely, miR-509-3p methylation was not observed in high miR-509-3p-expressing A2780 and OVCAR-3 cells (Fig. 3C). In addition, the cell invasion abilities of A2780CP70 and OVCAR-8 cells were significantly inhibited by 5-aza treatment (Fig. 3D).

We then examined the miR-509-3p methylation in EOC specimens. In EOC tissues, the promoter methylation site of miR-509-3p was detected in 54 (position 1, 33.5%), 53 (position 2, 32.9%), and 44 (27.3%) of the 161 ovarian samples at both positions. The frequency of methylation was significantly higher in EOC tumors with a low miR-509-3p expression (position 1, 48/79, 60.8%; position 2, 42/79, 53.2%) than in those with a high miR-509-3p expression (position 1, 6/82, 7.30%; position 2, 11/82, 13.4%, Table 2).

**Table 2:** The correlations between variables and M1 or M2 sites (*n* = 161)

			M1			M2		
		n	No	Yes	*P*	No	Yes	*P*
miR-509-3p	Low	79	31 (39.2)	48 (60.8)	< 0.001	37 (46.8)	42 (53.2)	< 0.001
	High	82	76 (92.7)	6 (7.3)		71 (86.6)	11 (13.4)	
COL11A1	Low	73	55 (75.3)	18 (24.7)	0.030	53 (72.6)	20 (27.4)	0.174
	High	88	52 (59.1)	36 (40.9)		55 (62.5)	33 (37.5)	
Age	< 52	67	50 (74.6)	17 (25.4)	0.064	52 (77.6)	15 (22.4)	0.016
	≥ 52	94	57 (60.6)	37 (39.4)		56 (59.6)	38 (40.4)	
Stage	Early	73	60 (82.2)	13 (17.8)	< 0.001	54 (74.0)	19 (26.0)	0.090
	Advanced	88	47 (53.4)	41 (46.6)		54 (61.4)	34 (38.6)	
Histology	Serous	94	57 (60.6)	37 (39.4)	0.064	63 (67.0)	31 (33.0)	0.985
	Non-serous	67	50 (74.6)	17 (25.4)		45 (67.2)	22 (32.8)	
Chemotherapy	CR/PR	99	67 (67.7)	32 (32.3)	0.236	74 (74.7)	25 (25.3)	0.253
	SD/PD	37	21 (56.8)	16 (43.2)		24 (64.9)	13 (35.1)	
PFI	≥ 6 m	124	89 (71.8)	35 (28.2)	0.009	87 (70.2)	37 (29.8)	0.128
	< 6 m	37	18 (48.6)	19 (51.4)		21 (56.8)	16 (43.2)	
Death	No	90	67 (74.4)	23 (25.6)	0.016	63 (70.0)	27 (30.0)	0.375
	Yes	71	40 (56.3)	31 (43.7)		45 (63.4)	26 (36.6)	

Data was presented as frequency (percentage)

Data was analyzed by Chi-square test or Fisher’s exact test

miR-509-3p level < 0.76 (low) and ≥ 0.76 (high); COL11A1 level < 1446.67 (low) and ≥ 1446.67 (high)

Additionally, the Kaplan–Meier curve, which was stratified by the promotor methylation site of miR-509-3p, showed that the patients with hypermethylation at position 1 had a significantly shorter OS (*P* = 0.019) and PFS (*P* = 0.005) than those without this hypermethylation. However, no survival differences were observed between those with or without hypermethylation at position 2 (Fig. 3E).

Cox regression model results indicated that the patients with hypermethylation at position 1 had a significantly higher risk of death (HR, 1.75; 95% CI, 1.09–2.80) and a significantly higher risk of progression (HR, 1.88; 95% CI, 1.21–2.93) than those without hypermethylation at position 1. Additionally, the patients with hypermethylation at both positions 1 and 2 had a significantly higher risk of progression (HR, 1.71; 95% CI, 1.06–2.77) and a higher risk of death (HR, 1.57; 95% CI, 0.94–2.60) than those without miR-509-3p promoter methylation. These results indicate that promoter hypermethylation might be the main cause of the downregulation of miR-509-3p in patients with EOC*.*

### Downregulation of miR-509-3p transcription by COL11A1 increases DNMT1 stability

**Fig. 4:**
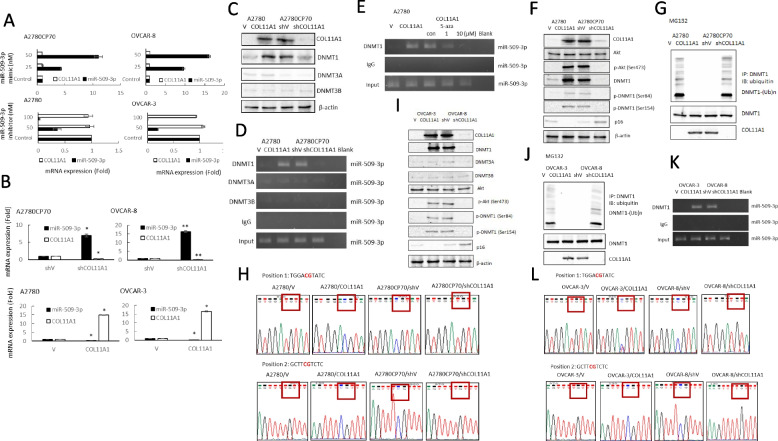
Downregulation of miR-509-3p transcription by COL11A1 increases DNMT1 stability. **A** The mRNA expression levels of COL11A1 and miR-509-3p in A2780CP70 and OVCAR-8 cells transfected with miR-509-3p mimics and in A2780 and OVCAR-3 cells transfected with the miR-509-3p inhibitor were evaluated using real-time RT-PCR. All experiments were performed in triplicate. **B** Upper panel: mRNA expression levels of miR-509-3p and COL11A1 in A2780CP70 and OVCAR-8 cells transfected with COL11A1 shRNA were evaluated using real-time RT-PCR. All experiments were performed in triplicate. * *P* < 0.05, ** *P* < 0.01, compared with shV. Lower panel: mRNA expression levels of miR-509-3p and COL11A1 in A2780 and OVCAR-3 cells transfected with the COL11A1 cDNA plasmid were evaluated using real-time RT-PCR. All experiments were performed in triplicate. * *P* < 0.05, compared with V. **C** A2780 cells were transfected with a COL11A1 cDNA plasmid and A2780CP70 cells were transfected with COL11A1 shRNA. COL11A1, DNMT1, DNMT3A, DNMT3B were evaluated using western blotting. β-actin was used as a loading control. **D** The binding activity of DNMT1, DNMT3A, and DNMT3B to the miR-509-3p promoter was evaluated using ChIP in COL11A1-overexpressing A2780 cells and COL11A1-knockdown A2780CP70 cells. Chromatin was isolated and immunoprecipitated using anti-DNMT1, -DNMT3A, and -DNMT3B antibodies, respectively. **E** The binding activity of DNMT1 to the miR-509-3p promoter was evaluated using ChIP in COL11A1-overexpressing A2780 cells treated with 1 or 10 µM 5-aza-2′-deoxycytidine for 5 days. Chromatin was isolated and immunoprecipitated using an anti-DNMT1 antibody. **F** A2780 cells were transfected with a COL11A1 cDNA plasmid and A2780CP70 cells were transfected with COL11A1 shRNA. The COL11A1, Akt, p-Akt (ser473), DNMT1, DNMT1 (ser84), DNMT1 (ser154), and p16 levels were evaluated using western blotting. β-actin was used as a loading control. **G** A2780 cells transfected with the COL11A1 cDNA plasmid and A2780CP70 cells transfected with COL11A1 shRNA and the corresponding cell lysates were immunoprecipitated using anti-DNMT1 antibodies. The resulting immunoprecipitates (IPs) were analyzed via immunoblotting (IB) using an anti-ubiquitin antibody. **H** Representative bisulfite sequencing of A2780 cells transfected with the COL11A1 cDNA plasmid and of A2780CP70 cells transfected with COL11A1 shRNA. **I** OVCAR-3 cells were transfected with a COL11A1 cDNA plasmid and OVCAR-8 cells were transfected with COL11A1 shRNA. The COL11A1, DNMT1, DNMT3A, DNMT3B, Akt, p-Akt (ser473), p-DNMT1 (ser84), p-DNMT1 (ser154), and p16 levels were evaluated using western blotting. β-actin was used as a loading control. **J** OVCAR-3 cells were transfected with a COL11A1 cDNA plasmid and OVCAR-8 cells were transfected with COL11A1 shRNA and the cell lysates were immunoprecipitated using anti-DNMT1 antibodies. The resulting IPs were analyzed via IB, using an anti-ubiquitin antibody. **K** The binding activity of DNMT1 to the miR-509-3p promoter was evaluated using ChIP in COL11A1-overexpressing OVCAR-3 cells and COL11A1-knockdown OVCAR-8 cells. Chromatin was isolated and immunoprecipitated using an anti-DNMT1 antibody. **L** Representative bisulfite sequencing of OVCAR-3 cells transfected with the COL11A1 cDNA plasmid and of OVCAR-8 cells transfected with COL11A1 shRNA

To further investigate the association between miR-509-3p and COL11A1, the mRNA expression levels of COL11A1 were analyzed using real-time RT-PCR in either miRNA-overexpressing or -inhibiting ovarian cancer cells. The COL11A1 expression did not change following the treatment with miR-509 inhibitor or miR-509 mimics (Fig. 4A). More interestingly, miR-509 expression increased in COL11A1 knockdown cells (Fig. 4B, upper panel) and decreased in COL11A1 overexpressing cells (Fig. 4B, lower panel), suggesting that COL11A1 is implicated in miR-509-3p regulation.

Mammalian DNA methylation is essential for development and is controlled by various factors, including 3 active DNA cytosine methyltransferases (DNMT1, DNMT3A, and DNMT3B), while DNMT1 is the main enzyme responsible for maintaining the methylation patterns [27]. An aberrant DNA methylation of CpG-island-containing promoters leads to gene silencing in both physiological and pathological contexts, especially in cancer cells [27]. Therefore, we hypothesized that the downregulation of miR-509-3p transcription by COL11A1 might be regulated by DNMT activation. First, we examined the expression of the three DNMTs in ovarian cancer cells with different COL11A1 expression statuses. COL11A1 depletion via RNA interference reduced the DNMT1 expression level in high COL11A1-expressing A2780CP70 cells. Conversely, the overexpression of COL11A1 increased DNMT1 expression in low COL11A1-expressing A2780 cells (Fig. 4C). However, the expression of DNMT3A and DNMT3B did not change. Furthermore, the binding of DNMT1 to the miR-509-3p promoter increased in COL11A1-overexpressing A2780 cells and decreased in COL11A1-knockdown A2780CP70 cells (Fig. 4D). The activity of the binding of DNMT1 to the miR-509-3p promoter was inhibited by 5-aza (Fig. 4E). There results indicate that COL11A1 downregulates miR-509-3p expression in ovarian cancer cells by inhibiting the binding of DNMT1 to the miR-509-3p promoter.

Increased phosphorylation of DNMT1 has been shown to influence DNMT1 stability [28]. We have previously demonstrated that COL11A1 confers chemoresistance to ovarian cancer cells through the activation and phosphorylation of Akt and phosphoinositide-dependent kinase-1 (PDK1) stabilization [13]. These results led us to hypothesize that the downregulation of miR-509-3p by COL11A1-mediated DNMT1 activation can be caused by an increased DNMT1 phosphorylation. Our results show that the phosphorylation of Akt and DNMT1 increased in COL11A1-overexpressing A2780 cells and decreased in COL11A1-knockdown A2780 cells (Fig. 4F). It has been shown that silencing DNMT1 lead to p16 activation [29]. The p16 decreased in COL11A1-overexpressing A2780 cells and increased in COL11A1-knockdown A2780 cells (Fig. 4F)**,** indicating p16 is another target gene influenced by DNMT1. After MG132 treatment, DNMT1 ubiquitination was more extensive in A2780/V cells than in A2780/COL11A1 cells, and it could be rescued by COL11A1 overexpression. Conversely, COL11A1 silencing facilitated the ubiquitination of DNMT1 in A2780CP70/shCOL11A1 cells (Fig. 4G). Sequencing analysis revealed that the miR-509-3p methylation pattern was obverse in A2780/COL11A1 and A2780CP70/shV cells, which showed a low miR-509-3p expression. Inversely, miR-509-3p methylation was not observed in A2780/V and A2780CP70/shCOL11A1 cells, which had a high miR-509-3p expression (Fig. 4H).

Similar phenomena were observed in OVCAR-3/COL11A1 and OVCAR-8/shV cells (Fig. 4I), whose expression levels of p-Akt and DNMT1 phosphorylation were increased. After MG132 treatment, the DNMT1 ubiquitination in OVCAR-3/V and OVCAR-8/shCOL11A1 cells was more extensive than in OVCAR-3/COL11A1 and OVCAR-8/shV cells (Fig. 4J). Furthermore, the binding of DNMT1 to the miR-509-3p promoter was increased in COL11A1-overexpressing OVCAR-3 cells and decreased in COL11A1-knockdown OVCAR-8 cells (Fig. 4K). The p16 expression decreased in COL11A1-overexpressing OVCAR-3 cells and increased in COL11A1-knockdown OVCAR-8 cells (Fig. 4K). Sequencing analysis revealed that the miR-509-3p methylation was obverse in OVCAR-3/COL11A1 and OVCAR-8/shV cells, which had a low miR-509-3p expression. Inversely, miR-509-3p methylation was not observed in OVCAR-3/V and OVCAR-8/shCOL11A1 cells, which had a high miR-509-3p expression (Fig. 4L). These results clearly indicate that COL11A1 promotes DNMT1 activation by increasing DNMT1 stability, which subsequently downregulates the miR-509-3p levels via hypermethylation.

### miR-509-3p targets SUMO-3 in ovarian cancer

**Fig. 5:**
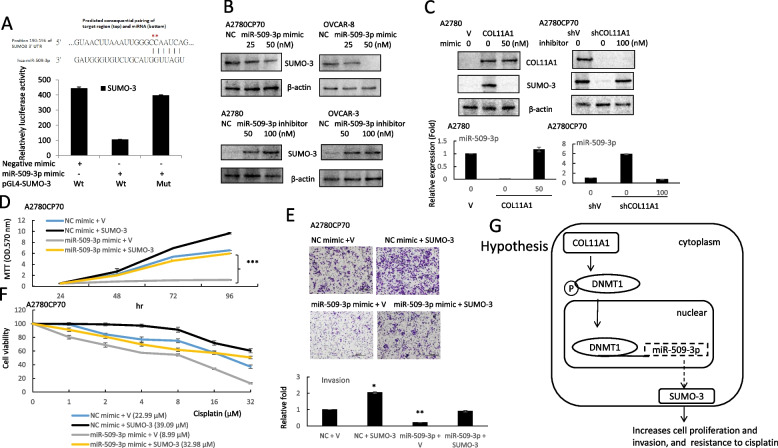
miR-509-3p regulates cell proliferation, invasion, and chemoresistance through SUMO-3. **A** Left panel: the putative miR-509-3p binding site in its 3′-UTR contains mutant wt-SUMO-3 and the corresponding mut-SUMO-3 (red star). Right panel: A2780CP70 cells were co-transfected with the wt-SUMO-3/mut-SUMO-3 vector and miR-509-3p NC/miR-509-3p mimics. Compared with that of the control group, the luciferase activity of the wt-SUMO-3 reporter gene was significantly reduced by miR-509-3p mimic transfection. In cells co-transfected with the miR-509-3p and mut-SUMO-3 reporter genes, the reporter gene activity did not significantly decrease. **B** The protein expression of SUMO-3 in A2780CP70 and OVCAR-8 cells transfected with miR-509-3p mimics, as well as that in A2780 and OVCAR-3 cells transfected with the miR-509-3p inhibitor, were evaluated using western blotting. β-actin was using as a loading control. **C** Upper panel: the protein expression of COL11A1 and SUMO-3 in A2780/COL11A1 cells transfected with miR-509-3p mimics, as well as that of A2780CP70/shCOL11A1 cells transfected with the miR-509-3p inhibitor, were evaluated using western blotting. β-actin was used as a loading control. Lower panel: the mRNA expression levels of miR-509-3p in A2780/COL11A1 cells transfected with miR-509-3p mimics, as well as those of A2780CP70/shCOL11A1 cells transfected with the miR-509-3p inhibitor were evaluated using real-time RT-PCR. All experiments were performed in triplicate. **D** A2780CP70 cells were co-transfected with pCMV3-ORF-SUMO-3 and miR-509-3p/NC. The MTT results showed that the cell proliferation rate was obviously decreased after transfection with the miR-509-3p mimics, while the overexpression of SUMO-3 reversed the apparent cell proliferation inhibition induced by miR-509-3p. NC: negative control; *** *P* < 0.001. **E** A2780CP70 cells were co-transfected with pCMV3-ORF-SUMO-3 and miR-509-3p/NC, and then their invasive ability was evaluated. All data represent the mean ± SD of three separate experiments; * *P* < 0.05, ** *P *< 0.01, compared with the control. **F** A2780CP70 cells were co-transfected with pCMV3-ORF-SUMO-3 and miR-509-3p/NC, and then treated with different concentrations of cisplatin for 48 h. Cell viability was assessed using 3-(4,5-dimethylthiazol-2-yl)-2,5-diphenyltetrazolium bromide assays. All experiments were performed in triplicate. **G** A model illustrating the hypothetical role of miR-509-3p regulation in ovarian cancer cells

Based on miRNA target analysis algorithms (miRanda and TargetScan), SUMO-3 is a potential target mRNA of miR-509-3p (Fig. 5A, upper panel). We demonstrated that the co-expression of miR-509-3p significantly inhibited the firefly luciferase reporter activity of the wild-type SUMO-3 3′-UTR, but not that of the mutant 3′-UTR (Fig. 5A, lower panel), using a dual-luciferase reporter system, indicating that SUMO-3 is a direct target of miR-509-3p.

Western blot analysis revealed that miR-509-3p overexpression significantly reduced the expression of SUMO-3 in A2780CP70 and OVCAR-8 ovarian cancer cells (Fig. 5B, upper panel), whereas miR-509-3p inhibitors noticeably increased the expression of SUMO3 in A2780 and OVCAR-3 cells (Fig. 5B, lower panel). Interestingly, the COL11A1-mediated increase in the SUMO-3 expression level in COL11A1-overexpressing A2780 cells was reduced via transfection with miR-509-3p mimics (Fig. 5C, left panel). The opposite effects were observed when COL11A1-knockdown A2780CP70 cells were transfected with the miR-509-3p inhibitor (Fig. 5C, right panel).

We further explored whether SUMO-3 was critical for the effect induced by miR-509-3p. The expression of SUMO-3 was inhibited by miR-509-3p, which could be rescued by the transfection of A2780CP70 cells with SUMO-3 (Figure S1). The MTT and invasion experiment results implied that the re-expression of SUMO-3 abolished the inhibiting influences of miR-509-3p on A2780CP70 cell growth (Fig. 5D) and invasion ability (Fig. 5E). SUMO-3 increased the cell resistance to cisplatin treatment (Fig. 5F). The increased cell sensitivity to cisplatin by miR-509-3p mimics was reduced by the re-expression of SUMO-3 (IC_50_ value: from 8.99 to 32.98 μM, *P* < 0.01, Fig. 5F). Therefore, miR-509-3p inhibited the aggressive phenotype of EOC cells by targeting SUMO-3.

## Discussion

miRNA dysregulation, which can result from aberrant DNA methylation, contributes to cancer tumorigenesis [30, 31]. In this study, we found that miR-509-3p expression was reduced in ovarian cancer, was mediated by the hypermethylation of the promoter region of the *miR-509-3p* gene, and significantly associated with a poor prognosis and COL11A1 expression. Mechanistic studies indicated that miR-509-3p transcription, which was downregulated by COL11A1 through an increased DNMT1 stability, was achieved by the binding of DNMT1 to the miR-509-3p promoter. miR-509-3p inhibited ovarian cancer cell growth, invasion ability, and enhanced their chemosensitivity by downregulating SUMO-3. The reinforced expression of SUMO-3 reversed the suppressive effects of miR-509-3p. These findings support the important roles of miR-509-3p in suppressing the tumorigenesis of ovarian cancer (Fig. 5G).

miR-509-3p is expressed from a genomic cluster of miRNAs of around 100 kb on chrXq27.3. The members of the Xq27.3 miR cluster have been reported to be strongly anti-correlated to a multi-cancer ‘metastasis-associated fibroblast’ gene signature [32]. Furthermore, the expression of these miR cluster members has been associated with the cancer stage and the survival of patients with ovarian cancer and is lower in omental metastases than in primary tumors [33–41]. miR-506-3p, a member of this miR cluster, is downregulated in pancreatic cancer due to the hypermethylation of its promoter region [42]. Therefore, we hypothesized whether the expression of other miRNAs in the cluster may be regulated by the same mechanism. In this study, we first performed sequencing to evaluate the detailed methylation patterns of two CpG islands in the promoter region of miR-509-3p in EOC cell lines and tumor samples and found that promoter hypermethylation by DNMT1 resulted in miR-509-3p silencing in ovarian cancer and that miR-509-3p hypermethylation was associated with a decreased survival time. miR-509-3p has been shown to target *CDK2* and to influence the cell cycle, colony formation, and migration of human lung and cervical cancer cell lines [43]. miR-509-3p directly targets *XIAP* to inhibit proliferation [11], and targets XIAP, Golgi phosphoprotein-3 (GOLPH3), and Wnt ligand secretion mediator (WLS) [9] to regulate platinum sensitivity in chemoresistant ovarian cancer cells. Another study showed that YAP1 is both a major effector of miR509-3p-mediated attenuation of migration and invasion, and spheroid formation in ovarian cancer cells [11]. Here, we provide evidence that a novel miR-509-3p epigenetic silencing alternation and SUMO-3 targeting reduce ovarian cancer cell aggressiveness and chemoresistance. SUMOs are a group of ubiquitin-like proteins that are attached to substrate proteins via a reversible post-translational protein modification termed SUMOylation. To date, five isoforms of SUMO have been identified in the human genome [44]. Dysregulated mRNA and protein levels of the SUMO machinery components have been implicated with certain prognosticators, such as a higher histological grade, a more advanced cancer stage, the presence of metastases, and a poor prognosis [44]. Further investigation is required to explore the exact mechanisms underlying the regulation of EOC cell aggressiveness and chemosensitivity by SUMO-3.

Our study suggests that a low miR-509-3p or miR-509-3p hypermethylation level is associated with a poor patient survival. Of the 161 EOC patients, the tumor miR-335 expressions of 137 patients have been described previously [24]. It is worth mentioning that the patients with low miR-509-3p and miR-335 levels had the shortest survival. Compared to patients with high miR-509-3p and miR-335 levels, the patients with low miR-509-3p and miR-335 levels tended to have shorter OS (Figure S2A, *P* < 0.001) and PFS (Figure S2B, *P* < 0.001). Similar findings were observed among patients with serous histology; compared to those with high miR-509-3p and miR-335 levels, those with low miR-509-3p and miR-335 levels had shorter OS (Figure S2C, *P* < 0.028) and PFS (Figure S2D, *P* = 0.058). Altogether, a low miR-509-3p level, especially when combined with a low miR-335 level, could help identify the patients with ovarian cancer at the highest risk of showing a poor clinical outcome. Therefore, miR-509-3p may serve as a prognostic biomarker of ovarian cancer.

We also provide evidence that miR-509-3p promoter hypermethylation, especially at position 1, may play a predictive role on the EOC prognosis. However, the occurrence rates of hypermethylation at position 1 or position 2 of the miR-509-3p promoter were diverse across different EOC histology. Most (81.8%) of the patients with mucinous histology had neither hypermethylation at position 1 nor at position 2 of the promoter of miR-509-3p (Supplementary Table 2). EOC is a highly heterogeneous disease. The mechanism of the progression or death of patients with mucinous EOC may be different from that of patients with other histological types. Further studies should be performed to collect more cancerous samples from patients with serous, endometrioid, or clear cell histology to validate these results.

DNMT1 dysregulation causes human diseases, such as cancer [45] and various genetic disorders [46, 47]. DNMT1 protein stability is reportedly regulated by methylation and phosphorylation [26, 48, 49]. DNMT1 phosphorylation has been shown to affect its methyltransferase activity [50] and its interaction with PCNA and UHRF1 [51], as well as DNMT1 stability [28]. Our previous report indicated that COL11A1 activates Akt phosphorylation [13]. Here, we showed that COL11A1 enhances DNMT1 protein stability (Figs. 4G and J). These results suggest that COL11A1 might regulate DNMT1 phosphorylation through Akt, and then induce the binding of DNMT to the miR-509-3p promoter (Fig. 4E and K). Our results also indicated that 5-aza treatment restored miR-509-3p expression (Fig. 3A) and reduced the cell invasion ability of EOC cells (Fig. 3D). Several epigenetic regulators, including DNMT inhibitors and histone deacetylase (HDAC) inhibitors, are currently under clinical investigation or have been approved for clinical use [52]. Our study provides new insight into an epigenetic therapeutic target for ovarian cancer treatment.

## Materials and methods

### Study population

This study adhered to the tenets of the Declaration of Helsinki and the research protocol was approved by the National Cheng Kung University Hospital Institutional Review Board (No. B-ER-107–396) and the Institutional Review Board of Chi Mei Medical Center (10808-L03)*.* Informed consent was obtained from patients. The patients with EOC who underwent primary cytoreductive surgery in the National Cheng Kung University Hospital and Chi Mei Medical Center, Liouying, and who had received front-line postoperative platinum-based chemotherapy between January 1, 2010, and December 31, 2016, were considered eligible to participate in this study. Non-cancerous patients who underwent partial oophorectomy for benign ovarian tumors were also included.

The cancerous and benign ovarian tissue samples were obtained intraoperatively and freshly stored in the vapor phase of liquid nitrogen (-196℃) or postoperatively collected from the NCKUH Biobank (Tainan, Taiwan). The medical records of patients were reviewed, and their clinical characteristics and information regarding the cancer stage, front-line chemotherapy, response to chemotherapy, and treatment outcomes were collected. Their follow-up records through October 31, 2021, were reviewed. The overall survival (OS) was calculated according to the date of diagnosis, and the progression-free survival (PFS) and PFI were determined based on the date of the last contact or progression following front-line chemotherapy.

### miRNA isolation

Ovarian cancer and non-cancerous (control) specimens were collected and de-identified. Total RNA was extracted from the specimens (100 mg with a tumor cellularity of 70% or greater), using a miRNeasy kit (Qiagen, Germany). The extracted RNA was quantified using a NanoDrop 1000 spectrophotometer (Thermo Scientific). In addition, the integrity of the extracted RNA was determined using an Agilent 2100 Bioanalyzer.

### Quantification of miR-509-3p

To obtain the miRNA distribution profile and quantify miR-509-3p in the specimens, we performed a quantitative real-time polymerase chain reaction (qPCR) according to the manufacturer’s instructions (Qiagen). In brief, 100 ng of total extracted RNA was collected and pooled from the samples, and cDNA was synthesized using a miScript Reverse Transcription kit (Qiagen). The cDNA sample was mixed with the qPCR master reagent (Human miScript Assay 384 set v10.1 [Qiagen]) using a Matrix Hydra eDrop (Thermo Scientific). Only wells with single melting-temperature values were included in further analysis. miRNAs were normalized with reference to the global miRNA mean and expression was calculated using the comparative C_t._ method. Statistical analysis was performed using the Student’s *t*-test. P-values < 0.05 were considered statistically significant.

### Quantitative reverse transcriptase PCR (RT-PCR)

The RNA obtained (5 μg) was used as the template for the cDNA synthesis reactions, together with random primers and superscript III reverse transcriptase (Applied Biosystems). The resultant cDNA solution (at a 1:20 dilution) was used to detect the level of the target gene mRNA using quantitative PCR (qPCR). An accurate quantitation was achieved based on standard curves, which were drawn by serially diluting a known amount of RNA obtained through an in vitro transcription reaction and by performing TaqMan qPCR using these dilutions, in addition to using the patient samples. Quantitative analysis of the mRNA expression was performed using the Light Cycler® 2.0 System (Roche Diagnostics GmbH). The primers and TaqMan probes used for the analyses were designed using the manufacturer’s software, Primer Express. The following primers were used: COL11A1 (HS01097664) and GAPDH (HS99999905). No-reverse-transcription (no-RT) control reactions were performed using 100 ng of the total RNA derived from each individual sample as a template to ensure that the amplification did not occur due to DNA contamination. No signal corresponding to the no-RT controls was detected. The target gene mRNA expression was assessed using real-time RT-PCR. *GAPDH* was used as an internal control for RNA quality. All quantitative analyses were performed in duplicate to assess the consistency of the results. The relative expression levels of the target gene, which were normalized to those of *GAPDH*, were calculated as follows: ΔC_t_ = C_t_(target) – C_t_(*GAPDH*). The ratio of the number of copies of the target gene mRNA to the number of copies of *GAPDH* was then calculated: 2^−Ct^ × K (K = 10^6^, a constant). Relative fold changes in gene expression were calculated using the comparative 2^−ΔΔCT^ method.

### Cells and media

The OVCAR-3 and OVCAR-8 cell lines were purchased from the National Cancer Institute DTP tumor repository program. The A2780 and A2780CP70 cell lines were provided by Dr. Hsu Keng-Fu (Department of Obstetrics and Gynecology, National Cheng Kung University Hospital, College of Medicine, National Cheng Kung University, Tainan, Taiwan). All cells were cultured in Roswell Park Memorial Institute-1640 medium supplemented with 10% fetal bovine serum and stored according to the manufacturer instructions. Cells used were passaged 5–20 times. Routine cell authentication was performed approximately every 6 months by cell morphology monitoring, growth curve analysis, species verification via isoenzymology, karyotyping identity verification via short tandem repeat-profiling analysis, and contamination checks. The most recent authentication was done in March 2022.

### DNA isolation and bisulfite sequencing analysis

Genomic DNA was isolated using a QIAamp DNA Mini Kit (Qiagen). Sodium bisulfite modification of the DNA was performed using an EZ DNA Methylation-Gold Kit (Zymo) according to the protocol of the manufacturer. The two CpG sites of the miR-509-3p promoter region were amplified via PCR using the bisulfite-modified DNA template. The methylated allele (M1) was amplified using the primers miR-509-3p-F (5′-GGTATAGAATATTTAGTATGTGG-3′) and miR-509-3p-R (5′-TTTCTATTTTATTTCTCTTTT-3′) and the methylated allele (M2) was amplified using the primers miR-509-3p-F (5′-AGGAAGAAAGAATAAGTTATTTA-3′) and miR-509-3p-R (5′-AAAACAATTA TTTCTTATATT-3′). The PCR product was analyzed using a BigDye Terminator cycle sequencing kit (Applied Biosystems, Foster City, CA) and an ABI 3730 automated capillary sequencer.

### Plasmid constructs and transfection

Small interfering RNAs (siRNAs) directed against human COL11A1 (sc-72956-SH), and a non-targeting negative control target (sc-108060) were purchased from Santa Cruz Biotechnology (Dallas, TX, USA). COL11A1 cDNA plasmid (BC117697, GE Healthcare) was cloned into a pCMV6-AC-GFP vector (PS100010, OriGene), followed by verification via sequencing. SUMO-3 (HG12782-UT) cDNA plasmid was purchased from Sino Biological (Beijing, China). miR-509-3p mimics (MC12984), mimics negative control (4464058), miR-509-3p inhibitor (MH12984), and inhibitor negative control (4,464,076) were purchased from Ambion (Foster City, CA, USA). A2780CP70 or OVCAR-8 cells were transfected with miR-509-3p mimics and SUMO-3 in combination using Lipofectamine 3000 (Thermo Fisher Scientific).

### Western blot analysis, antibodies, and reagents

Following protein extraction, equal amounts of protein were separated using 8–15% sodium dodecyl sulphate–polyacrylamide gel electrophoresis [12]. Antibodies against COL11A1 (GTX55142), DNMT1 (GTX116011), DNMT3A (GTX129125), and DNMT3B (GTX129127) were obtained from GeneTex (Irvine, CA, USA). An anti-β-actin antibody (sc-47778) was purchased from Santa Cruz Biotechnology (Dallas, TX, USA), whereas antibodies against Akt (9272), phospho-Akt (Ser473, 9271), ubiquitin (58,395), mouse IgG (7076), and rabbit IgG (7074) were obtained from Cell Signaling Technology (Danvers, MA, USA). An antibody against phospho-DNMT1 (Ser84) was purchased from Affinity Biosciences (Melbourne, Australia). An antibody against phospho-DNMT1 (Ser154) was purchased from Bioss Antibodies (Woburn, MASS, USA). An antibody against SUMO-3 was purchased from Abcam (Cambridge, UK). An antibody against p16 (AF5484) was purchased from Affinity Biosciences (Bath, UK). 5-aza-2′-deoxycytidine (5-aza) and MG132 were obtained from Sigma-Aldrich. Cisplatin (Fresenius Kabi Oncology, Ltd.) was provided by the Cancer Center of National Cheng Kung University Hospital.

### Cell proliferation and 3-(4,5-Dimethylthiazol-2-yl)-2,5-diphenyltetrazolium bromide (MTT) cytotoxicity assay

Cells (10^4^/well) were seeded on 96-well flat-bottomed microtiter plates, and then transfected with miR-509-3p mimics or miR-509-3p inhibitor and cultured for 24, 48, 72, and 96 h. For co-transfection, before miR-509-3p mimic treatment, the cells in the exponential growth phase were pretreated with SUMO-3 overexpression plasmids for 24 h and cultured for 24, 48, 72, and 96 h. For cisplatin treatment, after transfection with miR-509-3p or miR-509-3p inhibitor for 24 h, cells were treated with different cisplatin doses. After 48 h of incubation, the in vitro cytotoxic effects of these treatments were determined using an MTT assay (at 570 nm) and the cell viability was expressed as the percentage of control (untreated) cells (% of control). The MTT analysis was conducted as previously reported [12].

### Transwell invasion assay

The Transwell cell invasion assay was performed using polycarbonate membranes with 8 μm pores (Costar, Cambridge, MA, USA). Cells (5 × 10^4^) were seeded on the membrane of the upper chamber of the Transwell pre-coated with rat collagen I (60 µg/Transwell). Fibronectin in medium (0.6 mL) was added to the lower chamber as the chemoattractant in a 24 h assay at 37 °C under 5% CO_2_. The remaining cells in the upper chamber that did not migrate were removed using a cotton swab. The filters were fixed in 95% ethanol and stained with 0.005% crystal violet for 1 h. Migrated cells were counted using a phase-contrast microscope (Olympus, Lake Success, NY, USA). The mean of 10 contiguous fields represented the cell number. Each experimental condition was assayed in triplicate. used. The invasive capacity of cells was normalized to that of each corresponding control. One-sample unpaired Student's t-test was conducted to analyze the differences between the normalized invasive capacities obtained from the three independent experiments and the hypothetical value (which was set to 1).

### Luciferase reporter analysis

The SUMO-3 3′-UTR fragments with wild-type miR-509-3p binding sites (Wt) or mutated binding sites (Mut) were inserted into a pGL4 vector (Promega). The SUMO-3 3′-UTR PCR product was cloned into the *Sac*I/*EcoR*V site of the pGL4 vector. The following primers were used to target the SUMO-3 3′-UTR: forward, 5′-TTCACCACGATGATTTTCCT-3′ and reverse, 5′-GCACACAAAAGTACCCACAATATC-3′. The resultant construct was confirmed using DNA sequencing. Site-directed mutagenesis was performed to generate SUMO-3 3′-UTR constructs containing miR-509-3p mutant-binding sites by using the following complementary oligonucleotides: forward, 5′-CTGTAACTTAAATTGGGTTAATCAG-3′ and reverse, 5′-CTGATTAACCCAAT TTAAGTTACAG-3′. A2780CP70 or OVCAR-8 cells were transfected with the vector and the miR-509-3p mimics in combination. We performed luciferase assays 48 h post-transfection using a dual-luciferase reporter assay system (Promega). The normalized luciferase activity was reported as the ratio of luciferase activity to β-galactosidase activity. The activities of firefly luciferase and *Renilla* luciferase were measured as described previously [12].

### Chromatin immunoprecipitation (ChIP) assays

Native protein–DNA complexes were cross-linked via treatment with 1% formaldehyde for 15 min, and ChIP assays were performed as previously reported [13]. Briefly, equal amounts of isolated chromatin were subjected to immunoprecipitation using anti-DNMT1, anti-DNMT3A, anti-DNMT3B, and IgG monoclonal antibodies. Primers with the following sequences were used for the ChIP assays: miR-509-3p forward, 5′-GGTACAGAACATTCAGCATGTGG-3′ and reverse, 5′-AGAAAACTAGAAAAC TGTACAAA-3′.

### Statistical analysis

Data were analyzed using SPSS statistical software (version 21.0, IBM Corp., Armonk, NY, USA). Categorical variables are presented as frequencies and percentages and were analyzed using Chi-square test or Fisher’s exact test. Continuous variables are expressed as the mean ± standard deviation or as the median ± interquartile range. Interval variables were analyzed using Student’s t-test or Mann–Whitney U test. The cut-off values obtained based on the receiver operating characteristic curve for miR-509-3p, COL11A1 and miR-335 were optimized for their diagnostic sensitivity and specificity in predicting cancer progression or death. Survival was estimated using the Kaplan–Meier method and was compared using the log-rank test. Two-sided *P*-values < 0.05 were considered statistically significant. Cox proportional hazards models were implemented to estimate the hazard ratios (HRs) and 95% confidence intervals (CIs).

## Supplementary Information

**Additional file 1: Figure S1.** Left panel: the SUMO-3 protein expression in A2780CP70 cells co-transfected with pCMV3-ORF-SUMO-3 and miR-509-3p/NC was evaluated using western blotting. β-actin was used as a loading control. Right panel: the miR-509-3p expression in A2780CP70 cells co-transfected with pCMV3-ORF-SUMO-3 and miR-509-3p/NC was evaluated using real-time RT-PCR. All experiments were performed in triplicate.

**Additional file 2: Figure S2.** Ten-year OS (A) and PFS (B). Kaplan-Meier curves stratified by the miR-509-3p and miR-335 mRNA level and analyzed using by a log-rank test (*n* = 137). Ten-year overall survival (C) and progression-free survival (D) of the patients in the serous subgroups (*n* = 76). Kaplan-Meier curves stratified by the miR-509-3p and miR-335 mRNA level and analyzed using a log-rank test.

**Additional file 3: Figure S3.** Raw data.

**Additional file 4: Table 1.** EOC patient characteristics and the studied biomarkers (*n* = 161).

**Additional file 5: Table 2.** The correlations between variables and M1 or M2 sites (*n* = 161).

## Data Availability

The datasets used and/or analysed during the current study are available from the corresponding author on reasonable request.
