# Supplementary figures and images for: The downregulation of miR-509-3p expression by collagen type XI alpha 1-regulated hypermethylation facilitates cancer progression and chemoresistance via the DNA methyltransferase 1/Small ubiquitin-like modifier-3 axis in ovarian cancer cells
Source: J Ovarian Res. 2023 Jun 29;16:124. doi: 10.1186/s13048-023-01191-5 (PMC10308652; doi:10.1186/s13048-023-01191-5)

## Slide 1
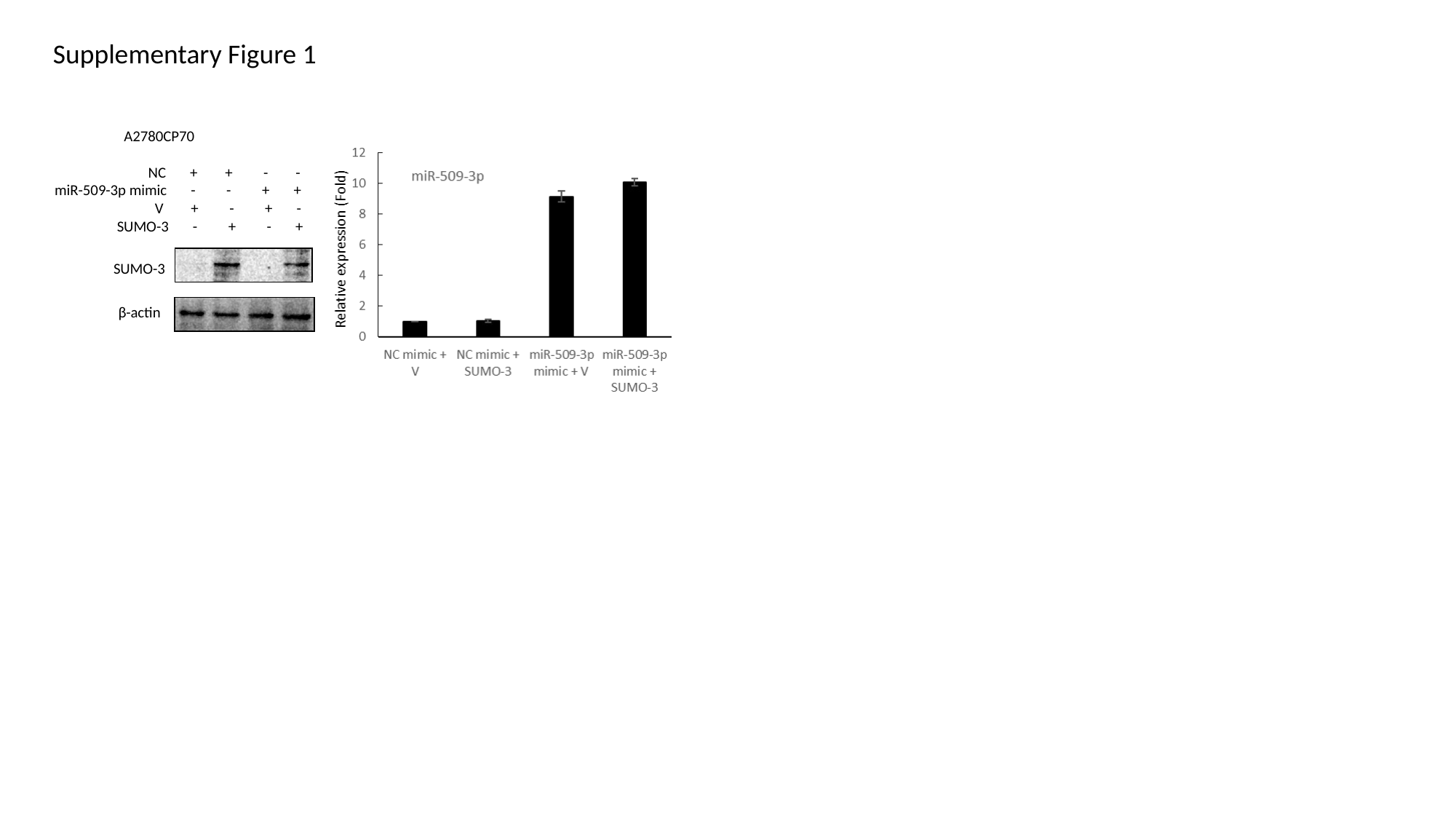

Supplementary Figure 1
 A2780CP70
 NC + + - -
 miR-509-3p mimic - - + +
 V + - + -
 SUMO-3 - + - +
SUMO-3
β-actin

Supplement: Supplementary file 1 — Additional file 1: Figure S1. Left panel: the SUMO-3 protein expression in A2780CP70 cells co-transfected with pCMV3-ORF-SUMO-3 and miR-509-3p/NC was evaluated using western blotting. β-actin was used as a loading control. Right panel: the miR-509-3p expression in A2780CP70 cells co-transfected with pCMV3-ORF-SUMO-3 and miR-509-3p/NC was evaluated using real-time RT-PCR. All experiments were performed in triplicate. [file 13048_2023_1191_MOESM1_ESM.ppt]
