# Supplementary material for: The downregulation of miR-509-3p expression by collagen type XI alpha 1-regulated hypermethylation facilitates cancer progression and chemoresistance via the DNA methyltransferase 1/Small ubiquitin-like modifier-3 axis in ovarian cancer cells
Source: J Ovarian Res. 2023 Jun 29;16:124. doi: 10.1186/s13048-023-01191-5 (PMC10308652; doi:10.1186/s13048-023-01191-5)
Supplement: Supplementary file 2 — Additional file 2: Figure S2. Ten-year OS (A) and PFS (B). Kaplan-Meier curves stratified by the miR-509-3p and miR-335 mRNA level and analyzed using by a log-rank test (n = 137). Ten-year overall survival (C) and progression-free survival (D) of the patients in the serous subgroups (n = 76). Kaplan-Meier curves stratified by the miR-509-3p and miR-335 mRNA level and analyzed using a log-rank test. [file 13048_2023_1191_MOESM2_ESM.ppt]

## Slide 1
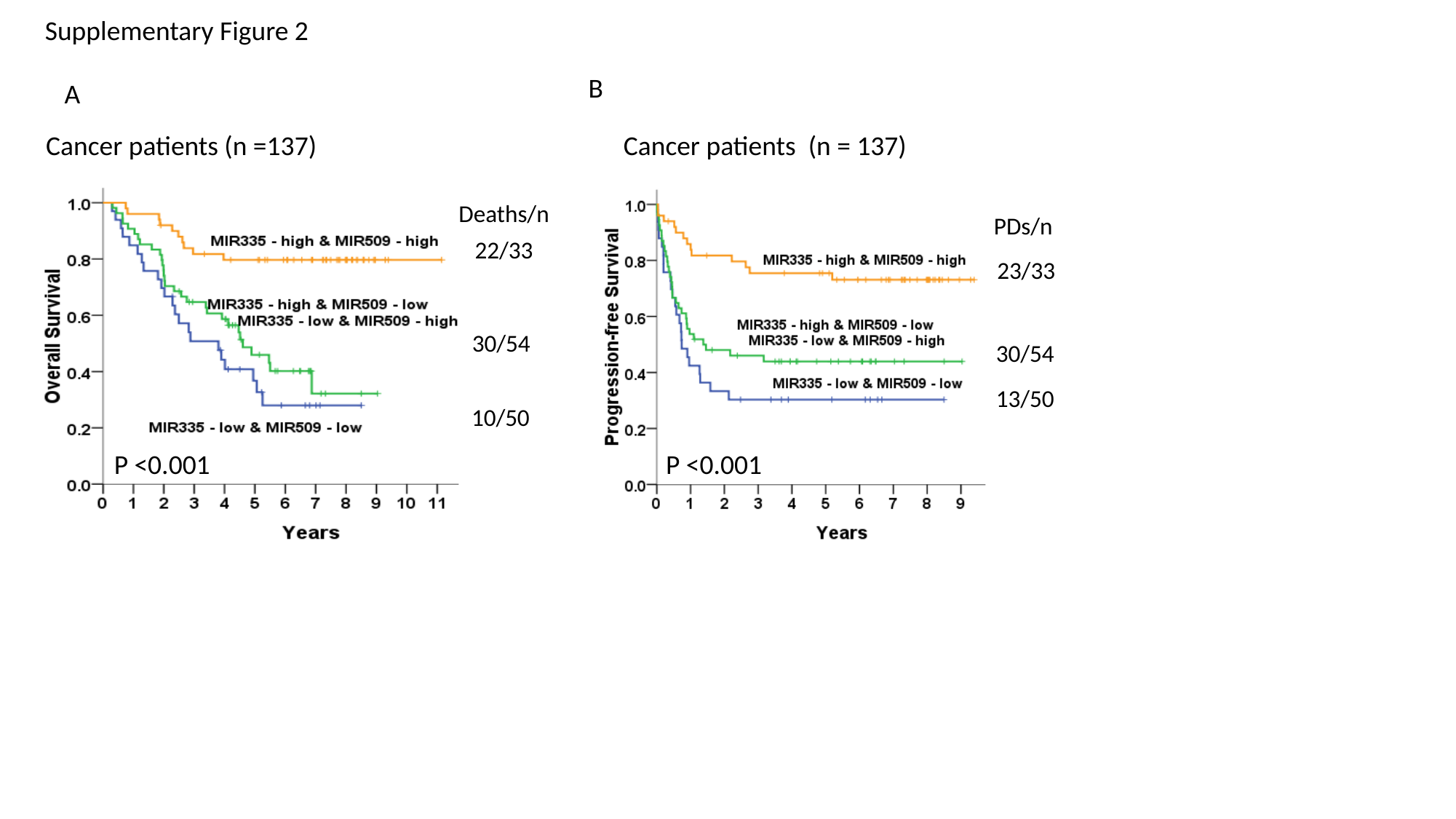

Supplementary Figure 2
B
A
Cancer patients (n =137)
Cancer patients (n = 137)
Deaths/n
PDs/n
 22/33
 23/33
 30/54
 30/54
 13/50
10/50
P <0.001
P <0.001

## Slide 2
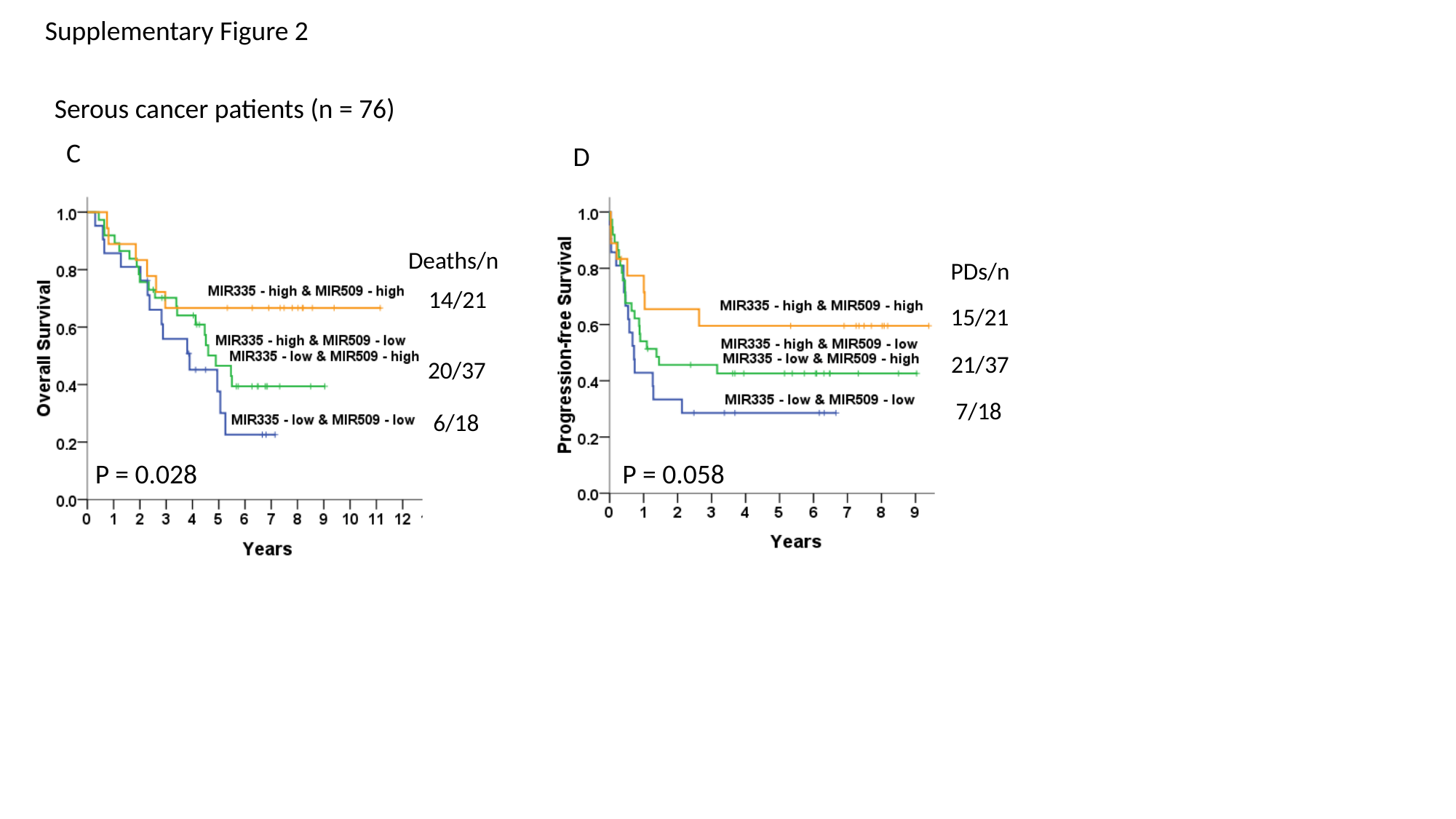

Supplementary Figure 2
Serous cancer patients (n = 76)
C
D
Deaths/n
PDs/n
14/21
15/21
21/37
20/37
7/18
6/18
P = 0.028
P = 0.058
