# Supplementary material for: The downregulation of miR-509-3p expression by collagen type XI alpha 1-regulated hypermethylation facilitates cancer progression and chemoresistance via the DNA methyltransferase 1/Small ubiquitin-like modifier-3 axis in ovarian cancer cells
Source: J Ovarian Res. 2023 Jun 29;16:124. doi: 10.1186/s13048-023-01191-5 (PMC10308652; doi:10.1186/s13048-023-01191-5)
Supplement: Supplementary file 3 — Additional file 3: Figure S3. Raw data. [file 13048_2023_1191_MOESM3_ESM.ppt]

## Slide 1
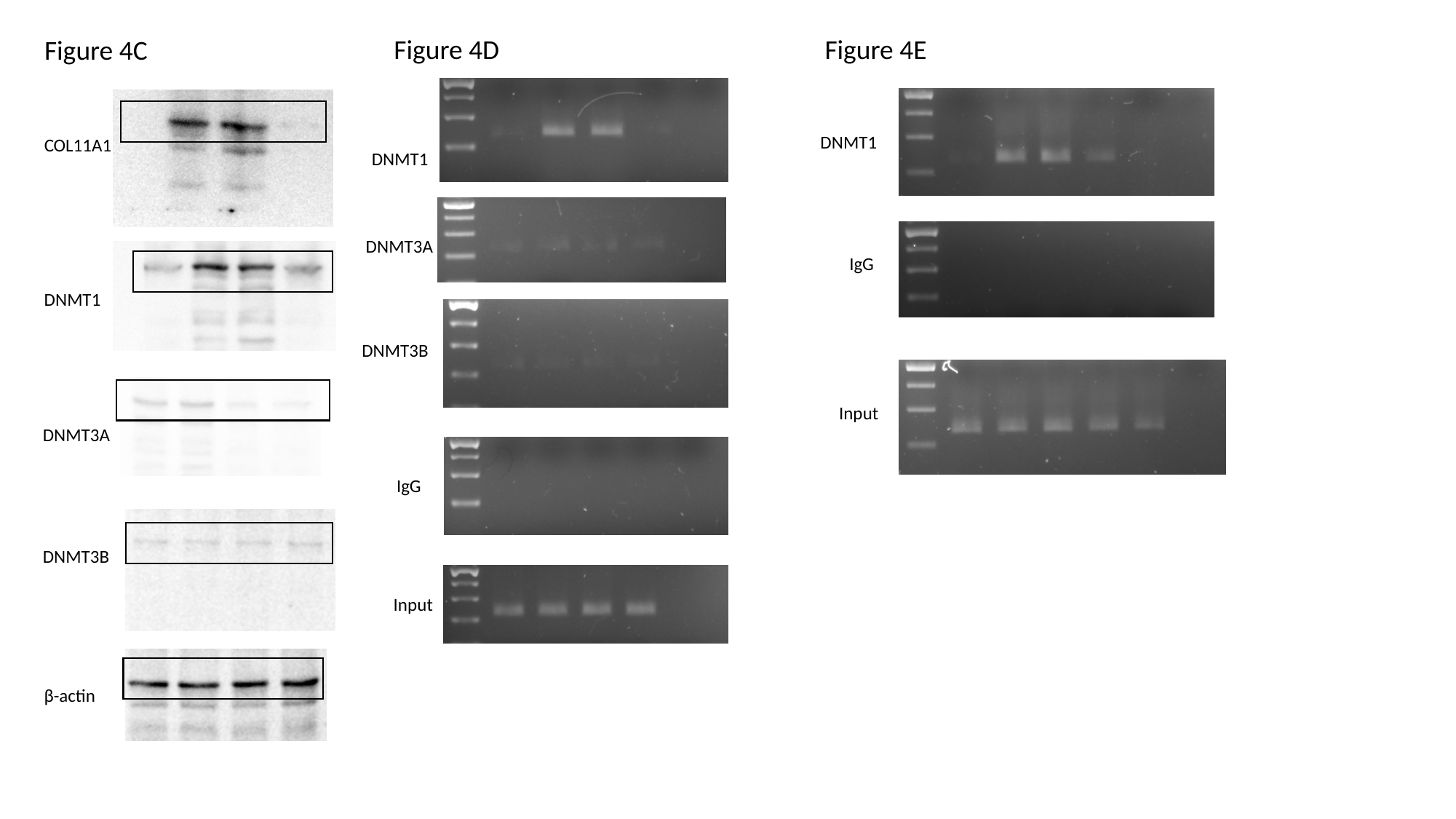

Figure 4E
Figure 4D
Figure 4C
DNMT1
COL11A1
DNMT1
DNMT3A
IgG
DNMT1
DNMT3B
Input
DNMT3A
IgG
DNMT3B
Input
β-actin

## Slide 2
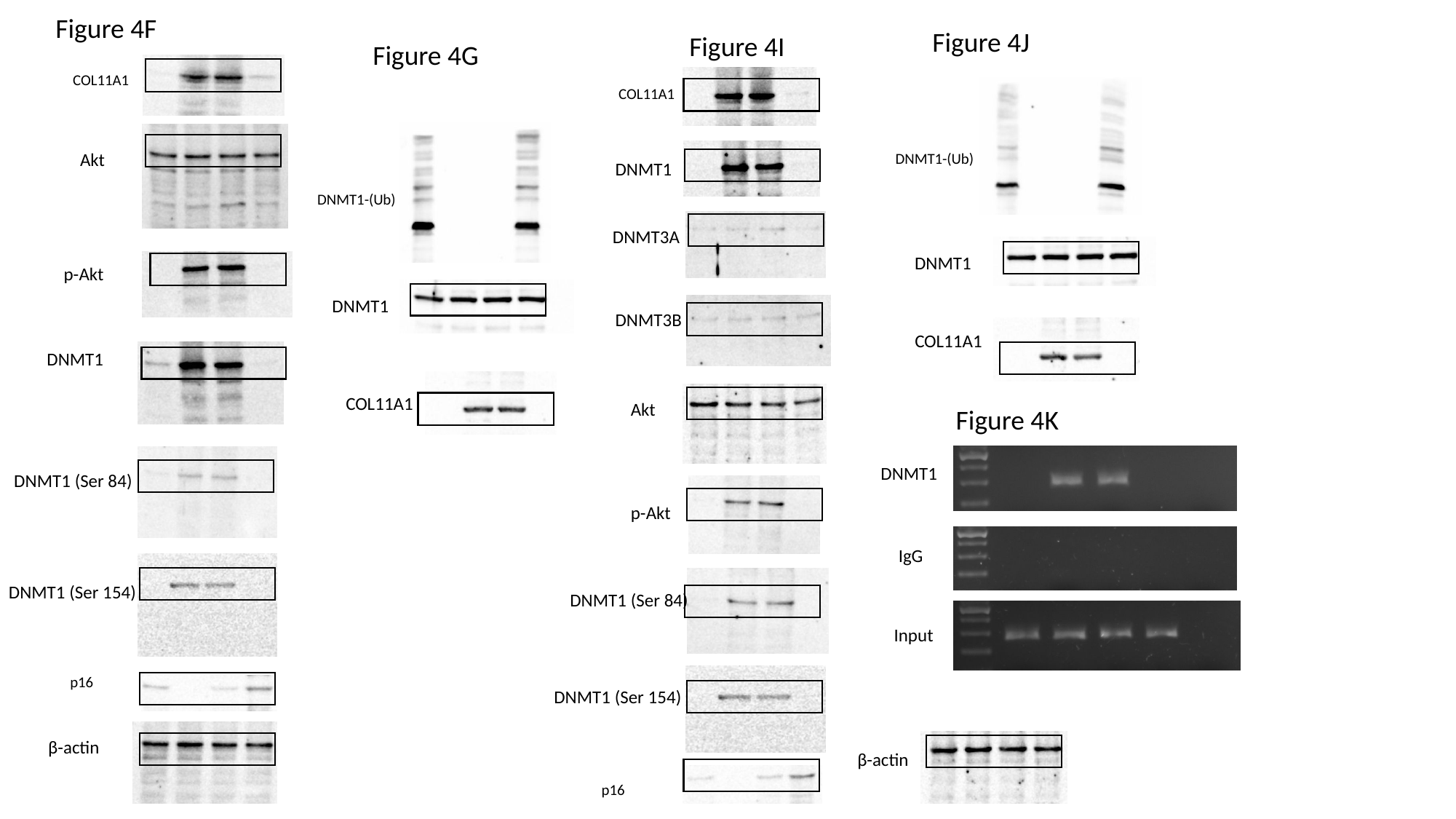

Figure 4F
Figure 4J
Figure 4I
Figure 4G
COL11A1
COL11A1
Akt
DNMT1-(Ub)
DNMT1
DNMT1-(Ub)
DNMT3A
DNMT1
p-Akt
DNMT1
DNMT3B
COL11A1
DNMT1
COL11A1
Akt
Figure 4K
DNMT1
DNMT1 (Ser 84)
p-Akt
IgG
DNMT1 (Ser 154)
DNMT1 (Ser 84)
Input
p16
DNMT1 (Ser 154)
β-actin
β-actin
p16

## Slide 3
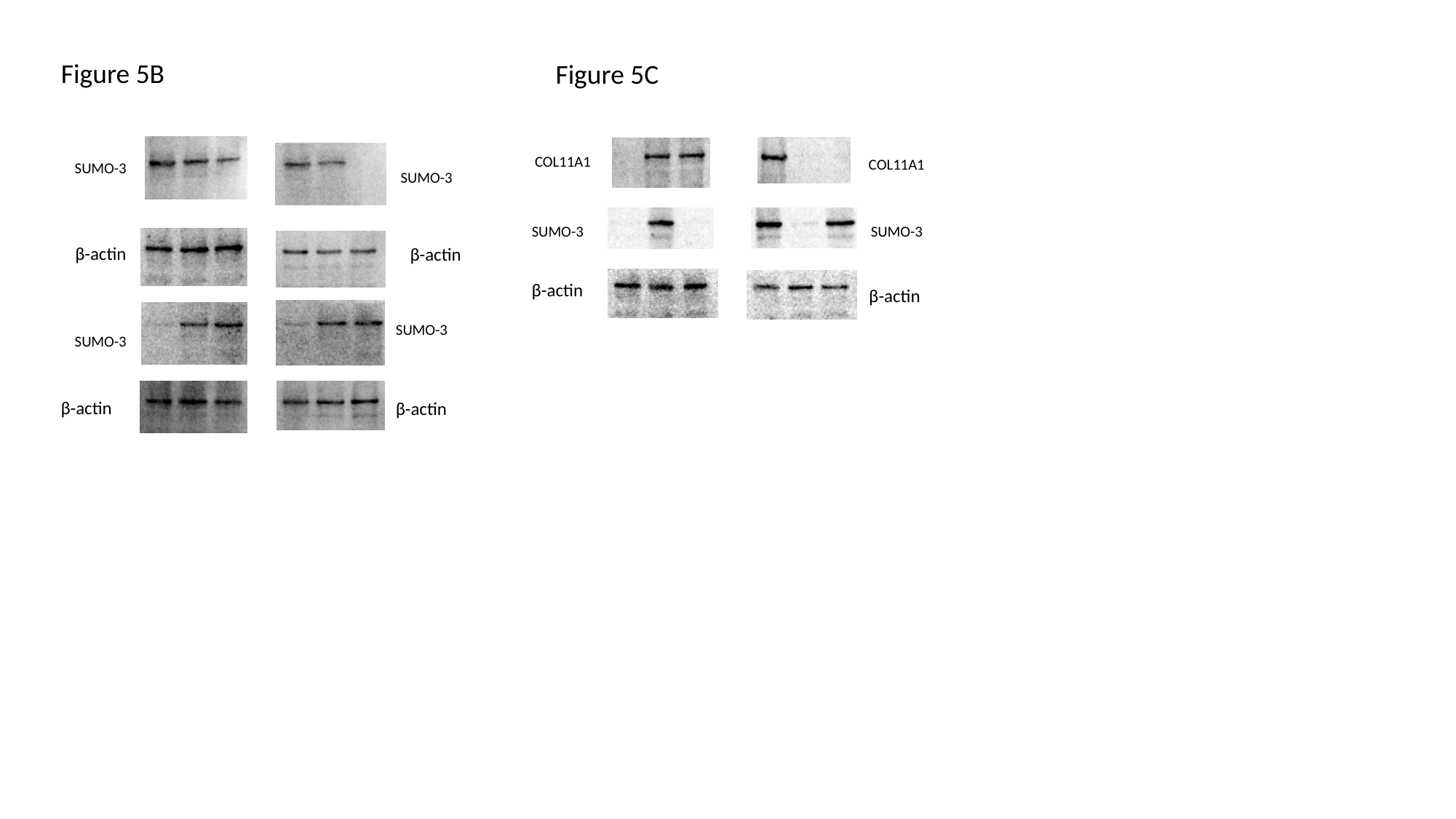

Figure 5B
Figure 5C
COL11A1
COL11A1
SUMO-3
SUMO-3
SUMO-3
SUMO-3
β-actin
β-actin
β-actin
β-actin
SUMO-3
SUMO-3
β-actin
β-actin
