# Supplementary material for: The downregulation of miR-509-3p expression by collagen type XI alpha 1-regulated hypermethylation facilitates cancer progression and chemoresistance via the DNA methyltransferase 1/Small ubiquitin-like modifier-3 axis in ovarian cancer cells
Source: J Ovarian Res. 2023 Jun 29;16:124. doi: 10.1186/s13048-023-01191-5 (PMC10308652; doi:10.1186/s13048-023-01191-5)
Supplement: Supplementary file 4 — Additional file 4: Table 1. EOC patient characteristics and the studied biomarkers (n = 161). [file 13048_2023_1191_MOESM4_ESM.doc]

**Supplementary Table 1. Correlations between different studied biomarkers.**

|  | | N | miR-509-3p | *P* | COL11A1 | *P* |
| --- | --- | --- | --- | --- | --- | --- |
| Non-cancer | | 23 | 242.45 ± 1939.42 | 0.09 | 0.58 ± 34.53 | <0.001 |
| Cancerous | | 161 | 0.77 ± 714.95 |  | 1784.83 ± 19051.66 |  |
| miR-509-3p | low | 79 | − |  | 10361.41 ± 44083.36 | <0.001 |
|  | high | 82 | − |  | 394.06 ± 9733.04 |  |
| COL11A1 | low | 73 | 317.71 ± 2685.18 | <0.001 | − |  |
|  | high | 88 | 0.46 ± 6.96 |  | − |  |

Data was presented as median ± interquartile range (IQR).

Data was analyzed by Mann-Whitney U test.

miR-509-3p level <0.76 ( low) and ≥0.76 (high); COL11A1 level <1446.67 ( low) and ≥1446.67 (high).
