# Supplementary material for: The downregulation of miR-509-3p expression by collagen type XI alpha 1-regulated hypermethylation facilitates cancer progression and chemoresistance via the DNA methyltransferase 1/Small ubiquitin-like modifier-3 axis in ovarian cancer cells
Source: J Ovarian Res. 2023 Jun 29;16:124. doi: 10.1186/s13048-023-01191-5 (PMC10308652; doi:10.1186/s13048-023-01191-5)
Supplement: Supplementary file 5 — Additional file 5: Table 2. The correlations between variables and M1 or M2 sites (n = 161). [file 13048_2023_1191_MOESM5_ESM.doc]

**Supplementary Table 2. Tumor histology and promoter methylation status of miR-509-3p (n = 161).**

|  | Serous  (n = 94) | Mucinous  (n = 11) | Endometrioid  (n = 11) | Clear cell  (n = 45) |
| --- | --- | --- | --- | --- |
| No M1 or M2 | 55 (58.5) | 9 (81.8) | 5 (45.5) | 29 (64.4) |
| M1 | 37 (39.4) | 0 () | 4 (36.4) | 13 (28.9) |
| M2 | 31 (33.0) | 2 (18.2) | 6 (54.5) | 14 (35.9) |
| Both | 29 (30.9) | 0 () | 4 (36.4) | 11 (28.2) |
